# Advanced interatrial block predicts recurrence of atrial fibrillation after accessory pathway ablation in patients with Wolff‐Parkinson‐White syndrome

**DOI:** 10.1002/clc.23222

**Published:** 2019-06-26

**Authors:** Jin‐Tao Wu, Dan‐Qing Zhao, Fei‐Fei Li, Rui Wu, Xian‐Wei Fan, Guang‐Ling Hu, Min‐Fu Bai, Hai‐Tao Yang, Li‐Jie Yan, Jing‐Jing Liu, Xian‐Jing Xu, Shan‐Ling Wang, Ying‐Jie Chu

**Affiliations:** ^1^ Department of Cardiology, Henan Provincial People's Hospital Henan Provincial People's Hospital of Henan University, Zhengzhou University People's Hospital, Central China Fuwai Hospital Zhengzhou China; ^2^ Department of Internal Medicine The Third Affiliated Hospital of Zhengzhou University Zhengzhou China

**Keywords:** accessory pathway ablation, advanced interatrial block, atrial fibrillation, Wolff‐Parkinson‐white syndrome

## Abstract

**Background:**

Paroxysmal atrial fibrillation (AF) frequently occurs in patients with Wolff‐Parkinson‐White (WPW) syndrome. Although successful ablation of the accessory pathway (AP) eliminates paroxysmal AF in some patients, in other patients it can recur.

**Hypothesis:**

We investigated the clinical utility of advanced interatrial block (IAB) for predicting the risk of AF recurrence in patients with verified paroxysmal AF and WPW syndrome after successful AP ablation.

**Methods:**

This retrospective study included 103 patients (70 men, 33 women; mean age, 44 ± 16 years) with WPW syndrome who had paroxysmal AF. A resting 12‐lead electrocardiogram was performed immediately after successful AP ablation to evaluate the presence of advanced IAB, which was defined as a P‐wave duration of >120 ms and biphasic [±] morphology in the inferior leads.

**Results:**

During the mean follow‐up period of 30.9 ± 20.0 months (range, 2‐71 months), 16 patients (15.5%) developed AF recurrence. Patients with advanced IAB had significantly reduced event‐free survival from AF (*P* < .001). Cox regression analysis with adjustment for the left atrial diameter and CHA_2_DS_2_‐VASc score identified advanced IAB (hazard ratio, 9.18; 95% confidence interval [CI], 2.30‐36.72; *P* = .002) and age > 50 years (hazard ratio, 12.64; 95% CI, 1.33‐119.75; *P* = .027) as independent predictors of AF recurrence.

**Conclusions:**

Advanced IAB was an independent predictor of AF recurrence after successful AP ablation in patients with WPW syndrome.

## 1 INTRODUCTION

Paroxysmal atrial fibrillation (AF) occurs frequently in patients with Wolff‐Parkinson‐White (WPW) syndrome, with a reported incidence of 9% to 38%.^1^, ^2^, ^3^, ^4^ Previous studies have reported a decreased incidence in AF after successful elimination of the accessory pathway (AP),^5^, ^6^ indicating that the AP itself may play an important role in the initiation of AF. However, paroxysmal AF still frequently recurs in some patients with WPW syndrome despite successful AP elimination.^3^, ^4^, ^7^, ^8^, ^9^ The identification of patients at high risk for recurrence of AF is of clinical importance because additional therapeutic strategies are needed for these patients.

Interatrial block (IAB) denotes a conduction delay between the right and left atria that manifests in a 12‐lead electrocardiogram (ECG) as a P‐wave duration of >120 ms.^10^, ^11^ A prolonged P‐wave with biphasic (±) morphology in the inferior leads represents an even higher degree of IAB and has been referred to as advanced IAB.^10^ The appearance of advanced IAB is frequently associated with atrial tachyarrhythmias, and has been found to predict AF in multiple clinical scenarios.^12^, ^13^, ^14^, ^15^, ^16^, ^17^, ^18^, ^19^, ^20^ However, the role of advanced IAB in predicting the recurrence of AF after AP ablation in patients with WPW syndrome is unclear. Thus, in the present study, we investigated the clinical utility of advanced IAB for predicting the risk of AF recurrence in patients with verified paroxysmal AF and WPW syndrome after successful AP ablation.

## 2 METHODS

### 2.1 Patients

Consecutive patients with overt or intermittent WPW syndrome who were hospitalized at Henan Provincial People's Hospital and Fuwai Central China Cardiovascular Hospital for radiofrequency ablation between January 2013 to September 2018 were retrospectively reviewed. The inclusion criteria were (a) at least one documented episode of AF before ablation; (b) performance of AP ablation alone, with no catheter ablation for AF; (c) successful catheter ablation, defined as the elimination of Kent bundle conduction by demonstration of atrial and ventricular pacing even after isoproterenol infusion; and (d) available records of a post‐ablation, 12‐lead ECG. The exclusion criteria were (a) repeated ablations; (b) previous cardiac surgery, congenital heart disease, or serious valvular heart disease; and (c) thyroid dysfunction on admission (abnormal free thyroxine or thyroid‐stimulating hormone level). The study protocol conformed to the ethical guidelines of the Declaration of Helsinki. All patients were informed about the investigational nature of the catheter ablation procedure and provided written informed consent to undergo the procedure. The study protocol was approved by the local institutional review board. The requirement for informed consent was waived because of the retrospective nature of the study.

### 2.2 Electrophysiological study and catheter ablation

The patients underwent an electrophysiological study after all antiarrhythmic drugs had been discontinued for at least five half‐lives and before radiofrequency catheter ablation was performed. Three 6‐French multipolar electrode catheters (Cordis Webster, Diamond Bar, California) were introduced percutaneously into the femoral veins for electrophysiological studies. The catheters were positioned in the high right atrium, across the tricuspid valve, to record the His‐bundle ECG, and in the right ventricular apex. Another 6‐French multipolar electrode catheter (Cordis Webster) was advanced from the right internal jugular vein and positioned in the coronary sinus. A retrograde aortic approach or the trans‐septal approach was used for left‐sided pathways. Right‐sided pathways were approached through the femoral veins. The radiofrequency current was delivered using a temperature‐controlled generator (GY‐8100; Huanan Medical, Zhengzhou, China) at 60°C. The locations of the APs were determined from the position of the catheter at a successfully ablated site in the left oblique fluoroscopic view.^21^

### 2.3 ECG analysis

**Figure 1:**
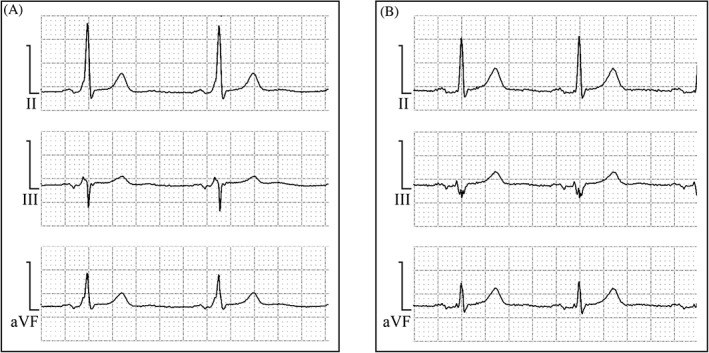
A, P‐wave morphology in a representative patient with Wolff‐Parkinson‐White syndrome in the inferior leads before accessory pathway (AP) ablation. B, Typical P‐wave morphology of advanced interatrial block with P‐wave duration >120 ms and biphasic (±) morphology in the inferior leads in the same patient after AP ablation

In all patients, a resting 12‐lead ECG in sinus rhythm (high‐pass filter, 0.05 Hz; low‐pass filter, 150 Hz; 25 mm/s; 10 mm/mv) was obtained immediately after the ablation procedure. All ECGs were transmitted electronically using Vhcloud Network Solution (Vales and Hills Biomedical Tech. Ltd., Beijing, China) for storage at the ECG Core Laboratory of Henan Provincial People's Hospital and Fuwai Central China Cardiovascular Hospital. ECGs were manually analyzed on a computer screen using digital calipers with scanning at 300 dots per square inch and 4‐fold image amplification. P‐waves were measured manually using digital calipers for all 12 ECG leads to identify the longest P‐wave duration, as previously described.^13^ Advanced IAB was defined as a P‐wave of >120 ms accompanied by a biphasic (±) morphology in the inferior leads (Figure 1).^10^ The ECG analysis was performed independently by two observers who were blinded to the patient details, and any differences between the observers were resolved by consensus.

### 2.4 Patient follow‐up

After the ablation procedure, all patients were required to visit their physician at 3, 6, and 12 months and every year thereafter. A 12‐lead ECG and 24‐hours Holter recording were obtained at every visit. If a patient exhibited any symptoms suggesting tachyarrhythmia, including palpitations, syncope, or dizziness, a new ECG and 24‐hours Holter recording were obtained. For any event reported between visit, the patient's medical records were retrieved and reviewed. All patients included in the study were followed up until occurrence of AF or until December 31, 2018 if no AF occurred. AF recurrence was defined as the occurrence of confirmed AF lasting more than 30 seconds as documented by ECG or Holter recordings.^22^

## 3 STATISTICAL ANALYSIS

All analyses were performed using statistical software (SPSS version 17.0; SPSS Inc., Chicago, Illinois). Continuous data are presented as mean ± SD and were compared using an unpaired independent‐samples *t*‐test or one‐way analysis of variance. Categorical variables are presented as a percentage of the group total and were compared using the χ^2^ test or Fisher's exact test as appropriate. A Kaplan‐Meier estimation with a log‐rank test was performed for unadjusted analysis of the association of advanced IAB with the risk of AF recurrence. Cox proportional hazards regression was used to examine the risk of recurrence. All probability values were two‐sided, and values of *P* < .05 were considered statistically significant.

## 4 RESULTS

In total, 103 patients were enrolled to the study. Advanced IAB was detected in 10 (9.7%) patients. The clinical and electrophysiological characteristics of patients with and without advanced IAB are shown in Table 1.The AP locations were single left‐sided AP in 60 (58.3%) patients, single right‐sided AP in 38 (36.9%) patients, and multiple APs in 5 (4.9%) patients.

**Table 1:** Characteristics of patients with and without advanced interatrial block

	All (n = 103)	aIAB (n = 10)	No aIAB (n = 93)	*P* value
Age, years	44 ± 16	67 ± 8	42 ± 15	<.001
Age > 50	41 (39.8%)	10 (100.0%)	31 (33.3%)	<.001
AF duration, months	9.0 ± 7.6	7.9 ± 6.0	9.1 ± 7.8	.649
Male, n (%)	70 (68.0%)	7 (70.0%)	63 (67.7%)	1.000
DM, n (%)	13 (12.6%)	5 (50.0%)	8 (8.6%)	<.001
Hypertension, n (%)	20 (19.4%)	5 (50.0%)	15 (16.1%)	.010
CAD, n (%)	7 (6.8%)	2 (20.0%)	5 (5.4%)	.278
CHA_2_DS_2_‐VASc score	0.8 ± 1.1	2.3 ± 1.1	0.6 ± 1.0	<.001
Left atrial diameter, mm	36.9 ± 4.2	43.1 ± 2.6	36.3 ± 3.8	<.001
LVEF, %	65.1 ± 5.4	64.8 ± 5.8	65.1 ± 5.4	.881
Ablation using the transseptal approach, n (%)	5 (4.9%)	3 (30.0%)	2 (2.2%)	.002
Electrophysiological characteristics				
Intermittent WPW syndrome, n (%)	10 (9.7%)	0 (0.0%)	10 (10.8%)	.597
Presence of retrograde conduction via AP, n (%)	100 (97.1%)	10 (100%)	90 (96.8%)	1.00
Antegrade ERP of AP	281 ± 42	274 ± 44	282 ± 41	.558
Single left‐sided AP, n (%)	60 (58.3%)	7 (70.0%)	53 (57.0%)	.649
Single right‐sided AP, n (%)	38 (36.9%)	3 (30.0%)	35 (37.6%)	.896
Multiple APs, n (%)	5 (4.9%)	0 (0.0%)	5 (5.4%)	1.00

Abbreviations: AF, atrial fibrillation; aIAB, advanced interatrial block; AP, accessory pathway; CAD, coronary artery disease; DM, diabetes mellitus; ERP, effective refractory period; LVEF, left ventricular ejection fraction; WPW, Wolff‐Parkinson‐White.

**Figure 2:**
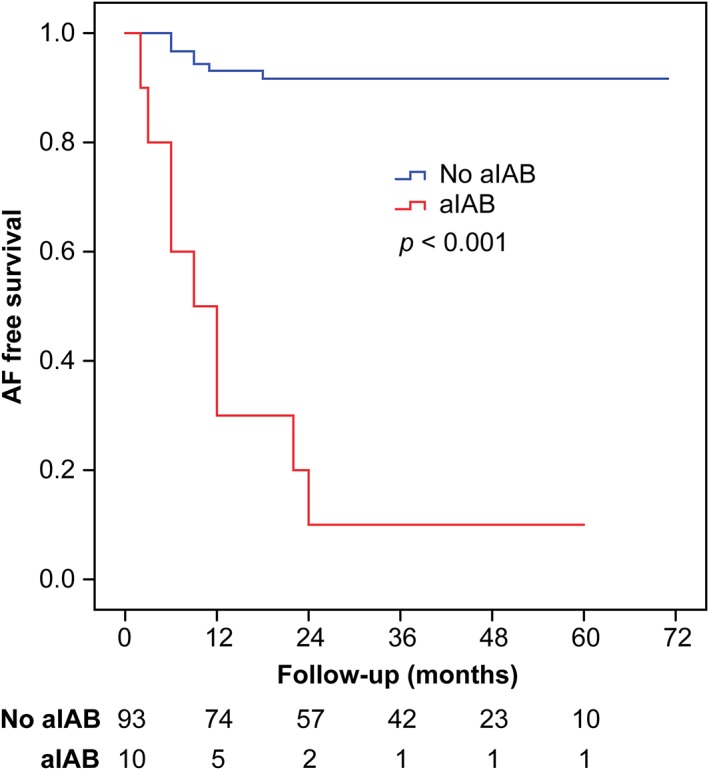
Kaplan‐Meier curves showing recurrence of atrial fibrillation (AF) in patients with and without a IAB) after accessory pathway ablation. Patients with advanced IAB had a higher rate of recurrence of AF than those without advanced IAB (90.0% vs 7.5%, respectively; *P* < .01 by log‐rank test). aIAB, advanced interatrial block

During the mean follow‐up period of 30.9 ± 20.0 months (range, 2‐71 months), 16 patients (15.5%) developed AF recurrence. The recurrence rate was 90.0% and 7.5% in patients with and without advanced IAB, respectively (log‐rank = 61.83, *P* < .001) (Figure 2). Patients with advanced IAB had a 12.0‐fold higher risk of AF recurrence than patients without advanced IAB. The characteristics of patients with and without recurrence are shown in Table 2. Patients with recurrence were older and had a higher CHA_2_DS_2_‐VASc score, larger left atrial diameter, and higher prevalence of advanced IAB than patients without recurrence.

**Table 2:** Characteristics of patients with and without recurrence of atrial fibrillation

	Recurrence (n = 16)	No recurrence (n = 87)	*P* value
Age, years	62 ± 9	41 ± 15	<.001
Age > 50	15 (93.8%)	26 (29.9%)	<.001
AF duration, months	8.3 ± 6.9	9.1 ± 7.8	.718
Male, n (%)	11 (68.8%)	59 (67.8%)	.941
DM, n (%)	4 (25.0%)	9 (10.3%)	.366
Hypertension, n (%)	6 (37.5%)	14 (16.1%)	1.000
CAD, n (%)	2 (12.5%)	5 (5.7%)	.656
CHA_2_DS_2_‐VASc score	1.9 ± 1.3	0.6 ± 0.9	.001
Left atrial diameter, mm	39.7 ± 5.5	36.4 ± 3.7	.004
LVEF, %	64.5 ± 6.1	65.2 ± 5.4	.664
aIAB, n (%)	9 (56.1%)	1 (1.1%)	<.001
Ablation using the transseptal approach, n (%)	2 (12.5%)	3 (3.4%)	.360
Electrophysiological characteristics			
ntermittent WPW syndrome, n (%)	1 (6.3%)	9 (10.3%)	.961
Presence of retrograde conduction via AP, n (%)	16 (100%)	84 (96.6%)	1.00
Antegrade ERP of AP	276 ± 47	282 ± 41	.595
Single left‐sided AP, n (%)	10 (62.5%)	50 (57.5%)	.708
Single right‐sided AP, n (%)	5 (31.3%)	33 (37.9%)	.611
Multiple APs, n (%)	1 (6.3%)	4 (4.6%)	1.00

Abbreviations: AF, atrial fibrillation; aIAB, advanced interatrial block; AP, accessory pathway; DM, diabetes mellitus; CAD, coronary artery disease; ERP, effective refractory period; LVEF, left ventricular ejection fraction; WPW, Wolff‐Parkinson‐White.

The univariate analysis identified significant differences in the number of patients aged >50 years, CHA_2_DS_2_‐VASc score, left atrial diameter, and incidence of advanced IAB in patients with and without AF recurrence. Thus, these factors were assessed using the Cox regression model. Cox regression analysis with adjustment for the CHA_2_DS_2_‐VASc score and left atrial diameter identified advanced IAB (hazard ratio, 9.18; 95% confidence interval [CI], 2.30‐36.72; *P* = .002) and age > 50 years (hazard ratio, 12.64; 95% CI, 1.33‐119.75; *P* = .027) as independent predictors of AF recurrence (Table 3).

**Table 3:** Multivariate analysis of predictors of atrial fibrillation recurrence after accessory pathway ablation

	*P*‐value	HR (95%CI)
Age > 50	.027	12.64 (1.33‐119.75)
Left atrial diameter	.316	0.92 (0.78‐1.08)
aIAB	.002	9.18 (2.30‐36.72)
CHA_2_DS_2_‐VASc	.531	1.16 (0.74‐1.81)

Abbreviations: aIAB, advanced interatrial block; CI, confidence interval; HR, hazard ratio.

## 5 DISCUSSION

The main findings of the present study are that advanced IAB and age > 50 years were independent predictors of AF recurrence after successful AP ablation in patients with WPW syndrome.

Previous studies have shown that patients with WPW syndrome have a high incidence of paroxysmal AF.^1^, ^2^, ^3^, ^4^ Recent studies suggest that although AP ablation alone may prevent further AF recurrence in the majority of these patients, the incidence of paroxysmal AF remains higher than that in the general population even after successful AP ablation.^3^, ^4^, ^7^, ^8^, ^9^ This is supported by our finding that 15.5% (16/103) of patients with verified paroxysmal AF and WPW syndrome developed AF recurrence after successful AP ablation. This phenomenon may be explained by the two mechanisms of paroxysmal AF in patients with WPW syndrome reported in previous studies.^9^, ^23^, ^24^ One mechanism involves AP‐dependent atrial electrophysiological abnormalities that are reversible, and the other involves AP‐independent atrial electrophysiological abnormalities that are intrinsic and seemingly irreversible even after successful AP ablation.

Previous studies have examined several predictors of AF recurrence in patients with WPW syndrome after AP ablation. Kawabata et al.^6^ investigated the relationship between the B‐type natriuretic peptide (BNP) level and AF recurrence after AP ablation in patients with WPW syndrome and found that a BNP level ≥ 40 pg/mL was an independent predictive factor for AF recurrence. However, the BNP level might fluctuate because it is affected by many factors. In a study by Hiraki et al.,^25^ a filtered P‐wave duration of >130 ms on signal‐averaged electrocardiography was an independent predictor of recurrence of AF after AP ablation. In addition, Aytemir et al.^26^ evaluated the predictive value of the maximum P‐wave duration and P‐wave dispersion on a 12‐lead surface ECG in predicting AF recurrence, and their multivariate analysis showed that only a P‐wave dispersion of ≥32.5 ms was an independent predictor of AF recurrence. In the present study, we evaluated the role of advanced IAB (ie, P‐wave duration of >120 ms accompanied by a biphasic (±) morphology in the inferior leads on 12‐lead surface ECG) in predicting the risk of AF recurrence in patients with WPW syndrome after AP ablation. The univariate and multivariate analyses showed that advanced IAB was associated with AF recurrence. Two potential mechanisms may account for these findings. First, advanced IAB is likely a presentation of AP‐independent atrial electrophysiological abnormalities that remain after successful AP ablation. In support of this, advanced IAB was previously reported to reflect the underlying atrial substrate with atrial fibrosis^27^, ^28^ and to be associated with the development of AF.^12^, ^13^, ^14^, ^15^, ^16^, ^17^, ^18^, ^19^, ^20^ Second, advanced IAB may play a role in initiating and maintaining reentry circuits by promoting the occurrence of unidirectional block,^29^ an important mechanism in the development of AF. Additionally, our study showed that age > 50 years was an independent predictor of AF recurrence, which is in accordance with the findings of previous studies.^3^, ^4^, ^7^

Because patients with advanced IAB have a high risk of AF recurrence after AP ablation, additional interventions are required to prevent AF recurrence in this population. Pulmonary vein isolation is an established effective treatment for paroxysmal AF.^30^ However, we did not assess whether pulmonary vein isolation was effective for preventing AF recurrence in patients in the present study. Although the pulmonary vein is reportedly involved in the development of paroxysmal AF in patients with WPW syndrome,^8^ atrial substrate abnormalities partially determine the likelihood of AF recurrence after AP ablation in these patients.^4^ Thus, the clinical efficacy of pulmonary vein isolation for preventing AF recurrence in these patients requires further study.

## 6 LIMITATIONS

This study has several limitations. First, it is not possible to correctly measure P‐wave duration before catheter ablation in patients with WPW syndrome because the delta wave overshadows the point at which the P‐wave ends; therefore, the use of P‐wave duration before ablation to predict the recurrence of AF in these patients is of limited value. Second, because all patients were retrospectively enrolled and underwent radiofrequency catheter ablation of AP using a two‐dimensional mapping system, detailed electrophysiological data such as intra‐atrial and interatrial conduction time and the details of electroanatomical mapping were not available. Further studies of prospectively enrolled patients are therefore required. Third, although we demonstrated that there was no anterograde AP conduction recurrence or atrioventricular reciprocating tachycardia recurrence in the AF recurrence group, it is possible that retrograde AP conduction was reestablished in these because we did not perform electrophysiologic studies during follow‐up. Thus, we cannot conclude that patients with AF recurrence had AP‐independent atrial electrophysiological abnormalities. Fourth, because the diagnosis of AF recurrence was based on clinical symptoms, ECG findings, and 24‐hours Holter recordings, the AF recurrence rate may have been underestimated because of a lack of symptoms in some patients. Additionally, P‐wave duration was measured based on the ECG obtained immediately after the ablation procedure; thus, residual atrial injury from the ablation procedure may have affected the P‐wave duration. Finally, the small sample size may have introduced statistical bias.

## 7 CONCLUSION

The presence of advanced IAB may be used to predict future recurrence of AF after successful AP ablation in patients with verified paroxysmal AF and WPW syndrome.

## CONFLICT OF INTEREST

The authors declare that there is no conflict of interest.
